# Will artificial intelligence improve residents’ quality of life without compromising healthcare quality? A pediatric point-of-view

**DOI:** 10.1186/s13052-025-02073-w

**Published:** 2025-10-01

**Authors:** Antonio Corsello, Francesco Pegoraro, Mattia Spatuzzo, Andrea Santangelo

**Affiliations:** 1 Department of Clinical-Surgical, Diagnostic and Pediatric Sciences, University of Pavia, Pavia, Italy; 2 National Association of Pediatric Residents (ONSP), Padua, Italy; 3 University of Milan, Milan, Italy; 4 Department of Experimental and Clinical Medicine, University of Florence, Florence, Italy; 5 Department of Maternal, Infantile and Urological Sciences, Sapienza University of Rome, Rome, Italy; 6 Department of Neurosciences, Rehabilitation, Ophthalmology, Genetics, Maternal and Child Health, University of Genoa, Genoa, Italy

**Keywords:** Pediatric residency, Artificial intelligence, ChatGPT, Predictive modeling, Clinical decision-making, Resident quality of life

## Abstract

**Background:**

The integration of artificial intelligence (AI) and advanced large language models in medical education and clinical practice is reshaping healthcare. These technologies have significant potential to enhance training experience and quality of life for medical residents. By automating routine tasks such as documentation and preliminary data analysis, AI-driven models can significantly reduce the workload, enabling residents to focus more on direct patient care and hands-on learning opportunities.

**Main Body:**

AI-driven support in diagnostics and decision-making may also reduce diagnostic errors, fostering a safer and more efficient healthcare environment. Furthermore, by alleviating administrative burdens, AI could play a critical role in mitigating resident burnout, contributing to a more resilient healthcare workforce and ultimately improving the continuity and quality of patient care. However, the adoption of AI in medical practice poses challenges. Automation risks reducing essential clinical skills, and over-reliance on AI may impact on professional autonomy and the development of diagnostic capacities. Concerns also persist regarding biased data, data security, legal issues, and the transparency in AI-driven decision-making processes.

**Conclusion:**

Addressing these challenges requires collaboration among healthcare professionals, AI developers and policymakers, as well as ethical frameworks and country-specific regulations. Only through a balanced and collaborative approach can we unlock AI’s full potential to create a more efficient, equitable, and patient-centered healthcare system.

## Background

In the evolving landscape of healthcare, the integration of artificial intelligence (AI) and advanced large language models (LLMs) in medical education and clinical practice presents significant opportunities and challenges [1]. The potential of these technologies is increasingly discussed, with significant implications for medical residents’ education and healthcare quality in the upcoming decades. LLMs and AI-driven tools are expected to improve diagnostic accuracy, enhance clinical decision-making, and workflow efficiencies, leading to better clinical outcomes and reduced stress for medical residents. On the other hand, AI poses critical challenges that must be addressed to ensure that residents’ education and clinical skills are not compromised. This paper focuses on residents, a unique hybrid between students and practicing physicians, who must constantly balance clinical responsibilities with educational development.

## Main text

### Opportunities in resident education

Medical residency is a period of intense learning, characterized by significant stress and workload. AI and LLMs, such as ChatGPT, developed for medical applications, offer the potential to alleviate some of these burdens. By assisting residents with various tasks, including administrative duties and complex diagnostic decisions, these tools have the potential to revolutionize daily operations in clinical settings. This transformation includes significant opportunities for data processing and scientific research, which can result in more time being devoted to clinical practice, but also improve research opportunities for early-career residents [2, 3].

A key advantage of AI language models is task automation. Automating documentation, patient history taking, and preliminary diagnostic assessments can save substantial time, allowing residents to focus more on patient care and hands-on learning [4]. This rapid processing of vast amounts of medical literature and individual patient data could in the next future provide evidence-based recommendations that support clinical decisions, improving efficiency and advancing the accuracy of diagnoses and treatments. One of the most promising applications of AI may lie in supporting differential diagnosis: AI can enhance diagnostic accuracy through its ability to synthesize vast datasets in real time. AI tools capable of simultaneously analyzing symptoms, patient history, and test results from large data sets can suggest appropriate diagnostic pathways, perform clinical evaluations and reduce errors due to human cognitive biases or time pressure. This support could be particularly valuable to clinicians who often work under high pressure and with limited experience, such as medical residents, conditions that contribute to higher rates of fatigue and stress. By minimizing diagnostic errors, AI can enhance safety and efficiency in healthcare systems, improving the quality of life for both clinicians and patients [5]. These factors could help in suggesting appropriate first-line tests and interventions in order to reduce times and costs spent on clinical evaluation, helping in managing health resources, especially in certain kinds of systems where either the wait time is extended, or complex diagnostic tools are hard to access. Some of these benefits could be specific for residents in Pediatrics, especially regarding decision-making in neonatal care or rare conditions, pediatric dosing complexities, or the development of AI-enhanced educational platforms.

Beyond clinical practice, AI-driven platforms offer tailored simulations and adaptive learning environments, providing opportunities to enhance residents’ technical and decision-making skills without increasing workload. Studies have shown that AI-powered educational tools significantly enhance clinical awareness, fostering practical learning opportunities in controlled settings [6].

**Fig. 1:**
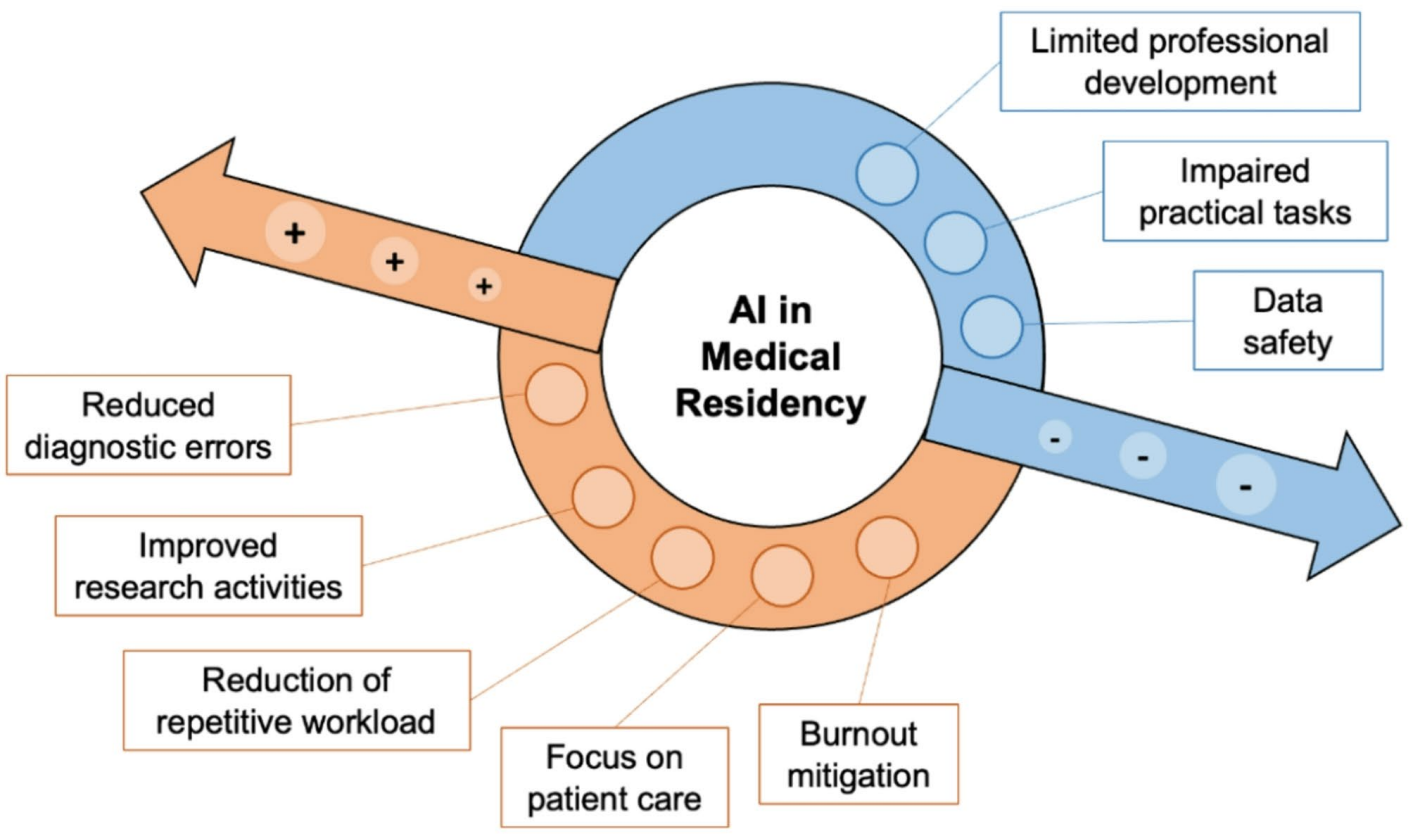
Advantages and limits of artificial intelligence (AI) in medical residency

Moreover, we should consider the long-term implications of AI in reducing burnout, which is extremely prevalent among medical residents [7, 8]. By offloading repetitive and time-consuming tasks to future approved and healthcare-specific LLMs, AI could help balance workload, allowing residents to focus on more meaningful clinical experiences and personal well-being. This change could foster a more resilient, engaged workforce and, ultimately, a higher standard of patient care (Fig. 1).

### Limits and challenges

Despite these benefits, the integration of AI in healthcare presents significant challenges and risks that must be addressed (Fig. 1). A primary concern is represented by the potential reduction in essential clinical skills among residents, such as patient history taking and physical examination, which are crucial for comprehensive patient care [9]. In fact, if these tasks become fully automated, residents may lose critical hands-on experience and the opportunity to develop necessary diagnostic skills. Additionally, the increased reliance of AI may diminish residents’ autonomy and decision-making capacity, potentially impairing their professional development. These risks apply to the whole spectrum of the educational path, which can be impaired by technological dependence, and by lack of creativity and focus [10].

AI systems are as good as their training data; hence, biased data may lead to biased outcomes. For instance, a study by Obermeyer et al. highlighted that AI algorithms might unintentionally reinforce systemic disparities, particularly in underserved populations, underscoring the need for diverse and representative training datasets [11]. This is particularly critical for Pediatrics, considering that AI models trained on adults might not be generalizable to children, which can be even more pronounced considering that rare diseases are more prevalent in children. Disciplines such as radiology to pathological anatomy, which rely heavily on visual recognition and extensive data collection, may be the first ones to ensure diverse and representative data sets, thereby preventing disparities in healthcare delivery. However, achieving this goal may take considerable time, as well as building trust among both healthcare professionals and patients, which requires enhancing the accuracy and transparency of AI’s decision-making processes. Moreover, since AI has become the area where several technology companies and health providers have been funding most financial investments in recent years, with this trend seems to be increasing, data security and privacy will be other crucial aspects to be discussed in the next future, raising issues of cybersecurity and the protection of sensitive data [12]. Indeed, it will be essential to ensure transparency in the decision-making processes of algorithms and to promote the training of doctors in the critical use of these technologies, with future specific regulations. In this regard, potential issues may also arise with regard of legal responsibility for AI-driven medical decisions. The absence of specific laws or regulations that address liability in the context of AI in healthcare, and regulatory principles were developed for humas, not algorithms. Potential grey areas that need to be addressed by healthcare-specific regulations include the potential responsibility of AI in medical errors, deontological questions, and the potential assimilation of AI to a medical device, which implies misfunctioning. The Italian healthcare lacks clear regulatory pathways, which mostly depends by EU initiatives as the latest National AI Strategy (2024–2026) is still in implementation stages. All these regulatory peculiarities of childhood care (parental jurisdiction and General Data Protection Regulation specificities) make this landscape additionally complex.

## Conclusions

The transformative impact of LLMs on residency programs and clinical practice presents opportunities. These technologies could soon enhance residents’ quality of life by reducing workload, improving diagnostic accuracy, and mitigating burnout, particularly in crisis situations such as pandemics. Careful and ethically conducted integration of AI in healthcare is essential to realize its full potential. Collaboration among healthcare professionals, AI developers, and policymakers will be essential for advancing AI-driven innovations. As we integrate AI into medical education, a key challenge will be finding a right balance. The success of AI in pediatric training will ultimately depend on our ability to design systems that complement, rather than replace, the unique strengths of human clinicians. By doing so, we can ensure that technology serves to empower the future of healthcare, rather than defining it.

## Data Availability

The datasets generated during and/or analyzed during the current study are available from the corresponding author upon reasonable request.

## Abbreviations

AI: Artificial intelligence
LLMs: Large language models
